# Mapping Avian Influenza Transmission Risk at the Interface of Domestic Poultry and Wild Birds

**DOI:** 10.3389/fpubh.2013.00028

**Published:** 2013-08-30

**Authors:** Diann J. Prosser, Laura L. Hungerford, R. Michael Erwin, Mary Ann Ottinger, John Y. Takekawa, Erle C. Ellis

**Affiliations:** ^1^Patuxent Wildlife Research Center, U.S. Geological Survey, Beltsville, MD, USA; ^2^Marine Estuarine Environmental Sciences, University of Maryland, College Park, MD, USA; ^3^University of Maryland School of Medicine, Baltimore, MD, USA; ^4^Patuxent Wildlife Research Center, U.S. Geological Survey, Charlottesville, VA, USA; ^5^Department of Animal and Avian Sciences, University of Maryland, College Park, MD, USA; ^6^Western Ecological Research Center, U.S. Geological Survey, Vallejo, CA, USA; ^7^Department of Geography and Environmental Systems, University of Maryland Baltimore County, Baltimore, MD, USA

**Keywords:** H5N1, avian influenza, spatial modeling, uncertainty, China, waterfowl, poultry, Monte-Carlo

## Abstract

Emergence of avian influenza viruses with high lethality to humans, such as the currently circulating highly pathogenic A(H5N1) (emerged in 1996) and A(H7N9) cause serious concern for the global economic and public health sectors. Understanding the spatial and temporal interface between wild and domestic populations, from which these viruses emerge, is fundamental to taking action. This information, however, is rarely considered in influenza risk models, partly due to a lack of data. We aim to identify areas of high transmission risk between domestic poultry and wild waterfowl in China, the epicenter of both viruses. Two levels of models were developed: one that predicts hotspots of novel virus emergence between domestic and wild birds, and one that incorporates H5N1 risk factors, for which input data exists. Models were produced at 1 and 30 km spatial resolution, and two temporal seasons. Patterns of risk varied between seasons with higher risk in the northeast, central-east, and western regions of China during spring and summer, and in the central and southeastern regions during winter. Monte-Carlo uncertainty analyses indicated varying levels of model confidence, with lowest errors in the densely populated regions of eastern and southern China. Applications and limitations of the models are discussed within.

## Introduction

Emerging infectious diseases in wildlife are a growing concern to human health. More than 75% of known emerging pathogens are zoonotic, or transmissible from animal to humans (1, 2). An increased global demand for meat products has caused rapid intensification of the domestic livestock industry (3), and improvements to transportation and market chains has brought humans and their agricultural systems closer together (4–6). Coupled with climate change and landscape fragmentation (7–9), incidence of emerging zoonoses is likely to continue to rise, with particular threat in developing regions such as China, India, and parts of Southeast Asia (7–10).

Two currently circulating avian influenza viruses, highly pathogenic A(H5N1) and low pathogenic A(H7N9) (hereafter H5N1 and H7N9) are of particular concern due to their high case-fatality rates (approximately 60 and 30% currently), and economic impact to the livestock industry and public health system (11). H5N1 first emerged in domestic geese in southern China in 1996 (12), and has since infected 60 countries across Asia, Africa, and Europe killing 374 people. It continues to persist with year 2013 reports of animal infection in Bangladesh, Bhutan, Cambodia, China, India, and Nepal (13) and human cases in Bangladesh, Cambodia, China, Egypt, Vietnam (11). H7N9 was first reported in a man from China who began showing symptoms in mid-February 2013. In the following 12 weeks, the number of human cases rapidly rose to 132 [as of 7 June 2013, (11)], all within China. At the time of this writing, human to human transmission is rare in both viruses, although concern exists that genetic mutation or reassortment could cause a human pandemic (14–16).

Wild birds, generally waterfowl and shorebirds (Orders Anseriformes and Charadriiformes), are the natural reservoir for low pathogenic avian influenza viruses (LPAIV) (17). LPAIV can mutate into lethal form, which commonly occurs upon entry into a high density host population, such as a poultry farm (18). This appears to have been the case with H5N1 (2), however, H7N9 remains an LPAIV (as defined by pathogenicity to chickens), despite having bridged the species gap and causing death in humans. Although many questions remain unanswered in these early months of H7N9 occurrence – such as which domestic or wild species are the reservoir for this virus – there is a growing body of knowledge from our H5N1 experience that might be applied toward understanding H7N9. In particular, understanding where wild and domestic birds have opportunity to interact on the landscape will be useful in identifying areas where disease transmission may be more likely to occur. These regions would become focal areas for surveillance and prevention.

A recent review of H5N1 risk models (19) noted that few studies have explicitly incorporated wild birds in transmission risk models, in part because obtaining adequate inputs for these populations is difficult. Our aim is to model regions where domestic and wild birds co-occur on the landscape, thereby presenting an opportunity for disease transfer. We take an iterative approach to understanding the spatial relationships between wild and domestic birds across the breeding and wintering seasons by building high spatial resolution (1 km) deterministic models based on wild and domestic bird co-occurrence, and subsequently by developing a model that incorporates H5N1 risk factors. The first set of models (co-occurrence) has broad utility for predicting areas for emergence of novel viruses, and potentially toward understanding H7N9. The second group of models provides insight on transmission potential at the domestic and wild bird interface for H5N1.

## Materials and Methods

### Study area

Transmission risk models were developed for China based on the importance of this region for H5N1 and H7N9, and the potential for emergence of novel viruses. China’s anthropogenic and natural landscapes differ greatly across the country, allowing for varying levels of disease risk, both spatially and temporally.

In addition to developing nationwide models for China, we focused on two areas of interest regarding transmission at the wild and domestic bird interface: Poyang Lake (PYL) Region in southeastern China and Qinghai Lake (QHL) in northwestern China. PYL, located in along the Yangtze River basin, is a complex wetland system that supports 8.8 million people, 14 million ducks, and 100,000 wintering migratory waterbirds, including 90% of the global population of endangered Siberian Cranes (*Grus leucogeranus*) (20). The majority of the human population at PYL lives in village settings, well-integrated within the agricultural landscape. Rice-cropping and free range duck farming are prevalent, and the demand for “healthy” wild meat has led to the rise of farmed wild waterfowl [Chinese spotbill (*Anas poecilorhyncha*), mallard (*Anas platyrhynchos*), northern pintail (*Anas acuta*), etc.], increasing the potential for wild and domestic populations to exchange virus (21, Cappelle, in review). QHL in contrast, is a remote arid region on the high-elevation Qinghai-Tibet Plateau with few poultry or free-ranging duck farms. Surprisingly, H5N1 outbreaks are common to both regions, and investigating the response using our transmission risk models to these very different regions is of particular interest.

### Model design and inputs

**Figure 1:**
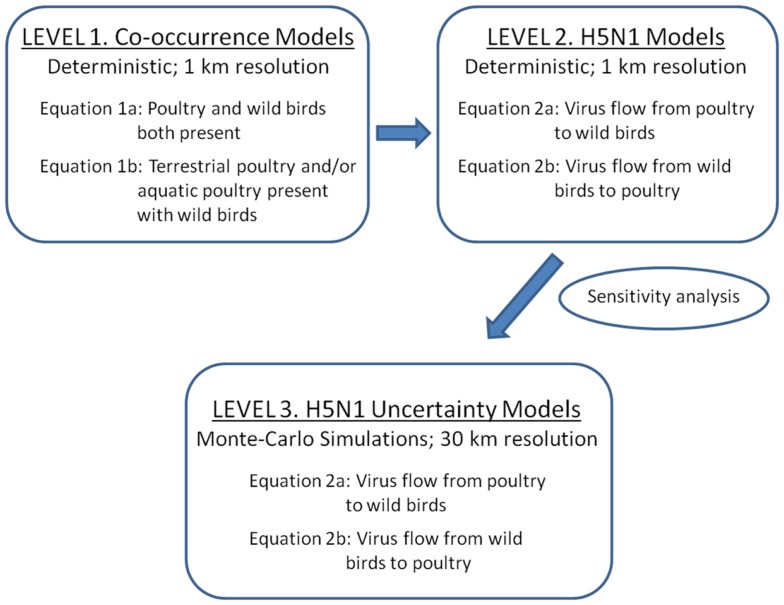
**Three levels of spatial models implemented for assessing H5N1 transmission risk between wild and domestic birds in China**. Level 1 and 2 deterministic models were developed to refine the transmission equations (1 km resolution). Level 1 models are co-occurrence models that predict where wild and domestic birds may come in contact. Level 2 models incorporate uni-directional equations for H5N1 transmission risk between poultry and wild birds. Level 3 models incorporate uncertainty using Monte-Carlo simulations at 30 km resolution.

We developed three levels of models to predict risk for disease transmission between poultry and wild waterfowl in China (Figure 1). As a first step (Level 1), we developed simple overlay gridded maps that predict where poultry and wild waterfowl are likely to co-occur on the landscape at 1 km resolution. Poultry is defined as aquatic poultry (domestic ducks and geese) and terrestrial poultry (chickens). Wild waterfowl is defined as wild duck and goose species, and does not include farmed wild birds. The Level 1 models show us where domestic and wild birds are likely to be present within close proximity (<1 km) to allow for potential disease transfer via direct transmission or the environment.

Level 2 and 3 models incorporate H5N1 parameters. The models include four basic components that relate to classic compartmental models in epidemiology (22): infected and susceptible populations and their viral shedding and exposure rates (see equations below). These models use a hybrid density-dependent and environmental transmission approach, whereby direct transmission is defined by bird to bird transmission (within poultry or waterfowl) as well as the fecal to oral route facilitated by transmission through the land and water environment (23, 24). We first use a deterministic approach to develop and refine the model equations (Level 2), then apply Monte-Carlo simulations to incorporate estimates of uncertainty around the parameter inputs (Level 3).

Nine parameters were derived for the risk models (Table 1), including spatial and non-spatial parameters: terrestrial poultry density, aquatic poultry density, effective waterfowl population, contaminant containment scalar, incoming biosecurity scalar, terrestrial poultry viral shedding rate, aquatic poultry shedding rate, effective waterfowl population shedding rate, and viral uptake rate. Spatial inputs include poultry and waterfowl densities and biosecurity scalars. Non-spatial inputs, meaning parameters for which the current state of knowledge does not allow for differentiation across the spatial extent of our model, include viral shedding and uptake rates. These parameters values, which were taken from the literature (Table 1), were applied uniformly across each grid cell in the model.

**Table 1:** **Parameters of 1 km resolution transmission risk equations including the range of values, approach for sensitivity analyses, and reference for each**.

Parameter	Description	Value range for 1 km (mean, SD)	Value range for 30 km	Notes (Reference)
*P*_te_	Terrestrial poultry density	0–9418 (379.4, 745.7) chickens/km^2^	0–5871 chickens/km^2^	Chicken densities for China (25 )
*P*_aq_	Aquatic poultry density	0–2796 (86.2, 164.7) ducks and geese/km^2^	0–2796 Ducks and geese/km^2^	Duck and goose densities for China (25)
*W*_EF_	Effective waterfowl population	*W*_EFbr_: 0–0.32 (0.01, 0.04)	*W*_prbr_: 0–0.29	Distributions from (Prosser, in review)
	Breeding season: *W*_EFbr_	*W*_EFwi_: 0–0.39 (0.006, 0.025) watefowl/km^2^	W_prw_ i 0 to 0.39	Population estimates from (43 , 44 )
	Wintering season: *W*_EFwi_			Prevalence rates from (45–49)
*C*_te_	Contaminant containment, terrestrial poultry (biosecure threshold *P*_te_ = 5000 birds/km^2^)	Biosecure = 0.75 and 0.25; non-biosecure = 1 (unitless)	Biosecure = 0.5; non-biosecure = 1	Biosecure threshold of 5000 chickens per km^2^. Reduction of population by 0.25 or 0.75 given biosecure designation
*B*_te_	Biosecurity, terrestrial poultry thresholds: *P*_te_ ≤ 50: *P*_backyard_ = *P*_te_ × 1.0 50 < *P*_te_ ≤ 1000, *P*_backyard:_ *P*_te_ × 0.5, *P*_te_ > 1000, *P*_backyard_ = 1000	*P*_te_ × *B*_te_ = 0–1000 poultry/km^2^	*P*_te_ × *B*_te_ = 0–1000 poultry/km^2^	Tri-part equation: At densities ≤ 50, 100% of population is backyard poultry From 50 to 1000, half are backyard poultry At greater than 1000, backyard poultry is limited to 1000
*V*_te_	Viral shedding rate, terrestrial poultry	10^1.4^ and 10^9.8^ EID_50_	10^0^, 10^9.8^, 10^6.8^ EID_50_	Viral shedding rates per individual per day from (50–52)
*V*_aq_	Viral shedding rate, aquatic poultry	10^1^ and 10^5.7^ EID_50_	0, 10^6.5^, 10^2.98^ EID_50_	Viral shedding rates per individual per day from (53–57)
*V*_wf_	Viral shedding rate, wild waterfowl	10^2.5^ and 10^6.5^ EID_50_	10^2.5^, 10^6.5^, 10^4.77^ EID_50_	Viral shedding rates per individual per day from (58)
*U*	Viral uptake = consumption rate of virus in the environment/minimum load for infection	10^−15^_/_(10^4.7^–10^1.8^) EID_50_	1.58e−17, 1.99e−20, 1.99e−20 ∑_50_	Consumption rate of virus in environment 10^-15^ (37); minimum viral load of 10^4.7^ EID_50_ (and 10^1.8^ EID_50_) to initiate infection with low pathogenic AIV (23, 59, 60)

We modeled terrestrial (*P*_te_) and aquatic poultry (*P*_aq_) populations by disaggregating census data and applying regression models for China’s top three poultry species: chickens, ducks, and geese (25, Prosser, in review). Poultry densities were modeled at 1 km resolution across the extent of China with units of birds per km^2^. For waterfowl, because the number of species and individual susceptibility to H5N1 is diverse (26), we aimed to derive an “effective waterfowl population” for each 1 km grid cell. Here we first modeled population estimates for each species by taking the total population for a given species and distributing it evenly across the extent of its range (Prosser, in review). We then applied prevalence rates [from the literature, see Table 1; (Prosser, in review)] to estimate the proportion of the population that may be (a) shedding virus if disease is present, or (b) susceptible to infection if disease is present in domestic species. To get the total effective waterfowl population (*W*_EF_), we then summed the resulting numbers of wild birds for each grid cell. Units for *W*_EF_ are birds per km^2^. This process was conducted separately for the breeding and wintering seasons (*W*_EFbr_ and *W*_EFwi_) as population distributions vary greatly due to the migratory patterns of waterfowl (Prosser, in review).

Two biosecurity scalars were developed to reduce the effective poultry populations: a contaminant containment parameter (*C*_te_) that moderates virus flow from poultry farms to the environment, and a biosecurity parameter (*B*_te_) that moderates exposure of poultry to virus [Table 1; (Prosser, in review)]. The parameters were derived based on the assumption that more biosecure farms control the flow of potential pathogens from the farm to the environment (*C*_te_ term), and protect themselves from incoming pathogens from the environment and other farms (*B*_te_ term); for example by cleansing vehicles before entering the farm, housing animals in structures secure from wild species, etc. Due to the difference in farming systems for chickens versus aquatic poultry [housed versus free range (27)], the biosecurity scalars were applied to the terrestrial poultry populations only. *C*_te_ was given a value of 1 for cells with less than 5,000 terrestrial poulty/km^2^ (i.e., no change in effective population) and 0.5 for cells with higher concentrations of birds (i.e., reducing the effective population by one half). *B*_te_ uses a tri-part scalar to reduce the effective population of chickens based on an estimate of the proportion of birds in backyard farms versus larger chicken houses [details in (28, Prosser, in review)]. The assumption made here is that if virus is transferred through the environment, only the free-ranging “backyard” poultry would be exposed to virus coming from wild birds. However, from our field studies, we also understand that movement of poultry feces and usage of water from different sources is complex and provides opportunity for virus transfer even to housed poultry. Therefore, we also ran the models using the entire poultry population as the effective population size (removing the *B*_te_ term from the model).

Virus shedding rates for terrestrial poultry (*V*_te_), aquatic poultry (*V*_aq_), and waterfowl (*V*_wf_) were taken from the literature (Table 1). Units are EID_50_ (amount of virus that causes infection in 50% of embryos) per individual per day. The virus uptake term (*U*), was taken from the literature (Table 1), and includes the consumption rate of virus in the environment (per day per bird) divided by the minimum load to infection (in units of EID_50_). The *U* term acts as a contact rate modifier for the number of susceptible birds in the population.

### Transmission risk equations

#### Level 1 models

We developed two Level 1 models, both of which are based on presence or absence of bird groups and not effective population numbers. The first model generated a simple binary risk map with the assumptions that (a) transmission risk is bi-directional (equal probability) between poultry and waterfowl and (b) transmission would only occur if both poultry and waterfowl were predicted to be “present”:

(1) $$T_{01A}=P_{01}\times W_{01}$$

where *P*_01_ and *W*_01_ are the predicted presence of poultry and waterfowl, respectively. Parameter values are 1 or 0 (present or absent). A *T*_01A_ value of 0 indicates no transmission risk (i.e., no co-occurrence) between domestic and wild species because both are not present within a cell. A transmission risk value of 1 indicates the potential for virus transmission between wild and domestic populations. The second Level 1 model measures the co-occurrence of waterfowl with one or both types of poultry. Transmission potential of these varying combinations may have implications for surveillance and control, which is why we included this iteration of the models. *Pt*_01_ is predicted presence of terrestrial poultry, and *Pa*_01_ is predicted presence of aquatic poultry. Parameter values are 1 or 0 (present or absent), and model output is 2, 1, or 0 represented in decreasing threat of transmission risk for the three output values (both types of poultry are present along with waterfowl; one type of poultry is present along with waterfowl; or either poultry or waterfowl are not present in the grid cell):

(2) $$T_{01B}=\left(Pt_{01}+Pa_{01}\right)\times W_{01}$$

#### Level 2 and 3 models

Level 2 and 3 models are based on where poultry and waterfowl are found together on the landscape, but also include effective population size, H5N1 shedding rates, and virus uptake for each group. We developed uni-directional equations for transmission potential from poultry to waterfowl versus waterfowl to poultry (Eqs 3 and 4) due to differences in farming structure and movement of virus through the environment (see above). The equations include compartments (grouped by brackets below) for the amount of virus entering the environment from infected birds (effective populations of infected birds times shedding rates) and amount of virus being taken up by susceptible individuals (effective susceptible populations times the uptake rate):

(3) $$\begin{array}{ll} T_{\text{P to W}} & =\left[\left(P_{\text{te}}\times C_{\text{te}}\times V_{\text{te}}\right)+\left(P_{\text{aq}}\times V_{\text{aq}}\right)\right]\times \left[W_{\text{EF}}\times U\right]\; \end{array}$$

(4) $$\begin{array}{ll} T_{\text{W to P}} & =\left[W_{\text{EF}}\times V_{\text{wf}}\right]\times \left[\left(\left(P_{\text{te}}\times B_{\text{te}}\right)+P_{\text{aq}}\right)\times U\right] \end{array}$$

where *P*_te_ and *P*_aq_ represent densities of terrestrial and aquatic poultry; *C*_te_ and *B*_te_ as biosecurity scalars; *V*_te_, *V*_aq_, and *V*_wf_ as viral shedding rates for terrestrial poultry, aquatic poultry, and waterfowl; *W*_EF_ as the effective waterfowl population; and *U* as the virus uptake rate. Model output is a measure of the transmission risk between poultry and waterfowl in units of predicted number of cases. Due to limits in the state of knowledge of shedding and uptake rates, we do not presume to use the model outputs as direct measures of the risk of transmission within a grid cell at this time; alternatively, we expressed the output in relative format by grouping the choropleth legend into 0 risk and 4 quantiles representing low, medium, high, and highest risk (29, 30). The Level 3 models used Monte-Carlo simulations to incorporate uncertainty around model inputs and to map estimates of error on a spatial basis (31, 32). Uncertainty for the poultry variables, *P*_te_ and *P*_aq_, was described using a normal distribution. This was determined by fitting a random sample of poultry estimates across 25 bootstrapped layers for 100 spatial locations [fitdistrplus package, R, (33, 34)]. We used best estimates and minimum – maximum limits within triangular and truncated normal distributions for the remaining variables [mc2d package, R, (34, 35)] to perform the triangular and truncated normal distributions. Each simulation was run for 10,000 iterations to ensure model convergence. We calculated the coefficient of variation (CV = standard deviation divided by the mean) to estimate uncertainty of model predictions using a bootstrap procedure for the poultry models (25) and Monte-Carlo analysis for the waterfowl indices (Prosser, in review).

### Spatial and temporal scale of analysis

The Level 1 and 2 deterministic equations were modeled at 1 km resolution in a geographic framework using ArcGIS 10.0 (ESRI, Redlands California) and Python (www.python.org). The Level 3 models, which incorporated the Monte-Carlo-based uncertainty (31, 32), were run at the coarser resolution of 30 km, approximately the average county size for China. Models were run separately for two temporal seasons relating the annual chronology of wild waterfowl to transmission risk: the breeding season (spring and summer months, generally April to July) and the wintering season (November to March).

## Results

### Risk models

**Figure 2:**
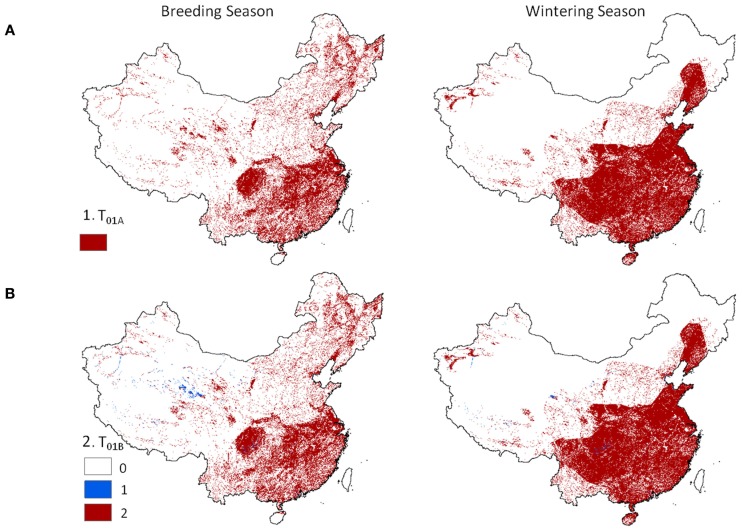
**Hotspot regions of potential disease transmission between domestic and wild birds in China**. Models are 1 km resolution co-occurrence for China’s domestic poultry and wild Anatidae waterfowl: **(A)** domestic poultry and wild Anatidae are predicted present (Eq. 1), and **(B)** terrestrial and aquatic poultry are predicted present in combination with wild Anatidae (red) versus presence of one poultry group (blue) with wild Anatidae (Eq. 2).

Level 1 models, identifying locations where poultry and wild waterfowl co-occur showed distinct patterns across China (Figure 2). Dense concentrations of positive risk grid cells were present across much of southeastern China during both seasons. Northeastern and western China showed more localized patterns of transmission risk, and wider extent for the breeding season than the wintering season. The spatial pattern was similar when considering the areas of co-occurrence of poultry with wild birds (Eq. 1) and presence of one or both poultry groups in combination with wild waterfowl (Eq. 2). Localized regions existed in the west where only one poultry type (usually chickens) was present in combination with wild waterfowl (Figure 2, lower panel).

**Figure 3:**
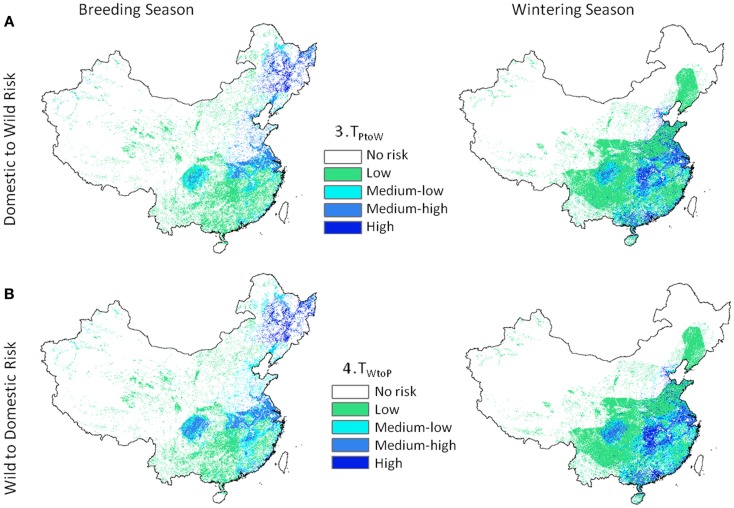
**Highly pathogenic H5N1 transmission risk between domestic poultry and wild Anatidae waterfowl at 1 km resolution for China**. Level 2 models include H5N1-specific transmission factors and are uni-directional with **(A)** representing transmission risk from domestic to wild birds (Eq. 3), and **(B)** from wild birds to domestic (Eq. 4).

**Figure 4:**
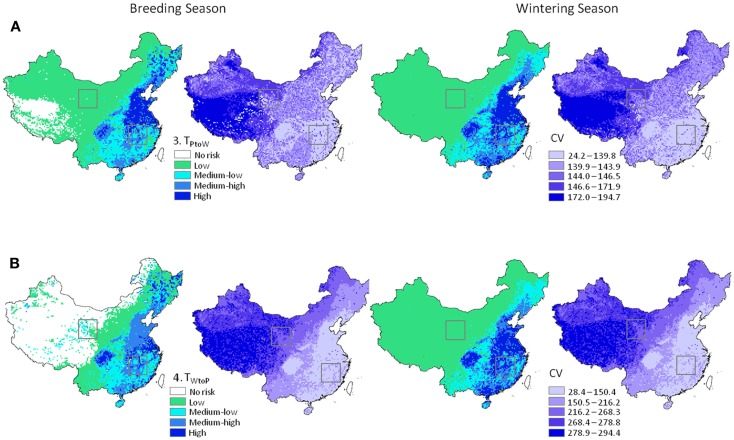
**H5N1 transmission risk between wild and domestic birds in China and associated uncertainty predictions at 30 km resolution**. Risk maps represented as mean and CV (left and right in each pair of maps, respectively). **(A)** Top panel represents transmission risk from poultry to wild waterfowl; **(B)** bottom panel represents transmission risk from wild waterfowl to poultry. Maps are symbolized using quantiles. Black boxes correspond to the Qinghai Lake and Poyang Lake Regions outlined in Figure 5.

**Figure 5:**
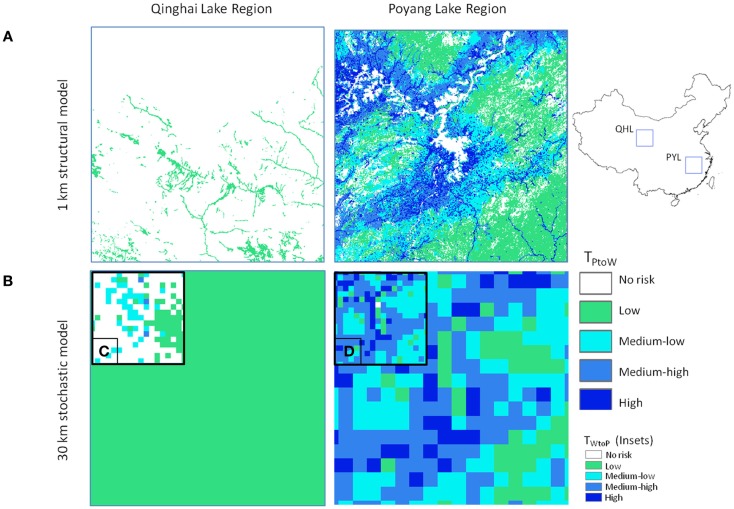
**Comparison of model outputs for Qinghai Lake (QHL) and Poyang Lake (PYL) sub-regions for (A) 1 km deterministic and (B) 30 km Monte-Carlo model outputs using Eq. 3 (poultry to wild transmission risk) for the winter season**. Insets **(C,D)** show comparisons for the breeding season Eq. 4 (wild to poultry transmission).

Transmission risk for the 1 km deterministic poultry to wild equations (Eq. 3, *T*_P__to__W_) ranged from 0 to 164.8 e−9 with units of predicted number of cases per day. Wild to poultry deterministic transmission risk was lower (0 to 5.19 e−11). Ranges for the 30 km Monte-Carlo models were 0 to 67.0 e−9 and 0 to 16.3 e−12 for Eqs 3 and 4, respectively. The mean level of risk was greater for the poultry to waterfowl models (Eq. 3) by approximately two orders of magnitude (Table 2). Spatial patterns of disease risk were similar across the broad scale of China (Figure 3). Both the 1 km deterministic and 30 km Monte-Carlo models showed distinct spatial patterns between seasons (Figures 3 and 4, respectively). During the breeding season, highest levels of risk (in both directions) were localized patches in northeastern China and along the Yangtze River plain of south-central China. For the wintering season, higher levels of risk were confined to southern and eastern China, particularly along the major river basins. Winter models had higher means than breeding season models by 46% Eq. (3) and 53% Eq. (4). Focused model predictions for the PYL and QHL sub-regions showed contrasting results between seasons (Figure 5). PYL had moderate to high transmission risk in both the winter and summer seasons whereas QHL showed low risk during the winter season and moderate risk during the summer breeding season.

**Table 2:** **Differences in mean values across all cells for two modeling approaches (Level 2 and 3) and four transmission scenarios**.

Model	Eq. 3 breeding season	Eq. 3 wintering season	Eq. 4 breeding season	Eq. 4 wintering season
Level 2 (deterministic)	3.82E−10	7.13E−10	1.48E−13	3.13E−13
Level 3 (Monte-Carlo)	1.18E−09	1.66E−09	6.03E−13	8.39E−13
Coefficient of variation	144	147	219	223

*Units of Level 2 and 3 model output are predicted number of cases per day*.

Patterns in uncertainty of the Level 3 Monte-Carlo simulation models were similar across seasons and uni-directional equations. We carefully investigated mean input and output for the models between the Level 2 deterministic and Level 3 Monte-Carlo simulations (36). Means were nearly identical for the 1 and 30 km models, indicating a lack of bias in the resampling process (Table 3); we also noted higher means for the input parameters that were modeled using the triangular distribution of the Monte-Carlo models (Table 3, Section B). These models initially used a global (fixed) minimum and maximum value for all cells which is less computationally intensive but has the effect of increasing the mean values. We then reran the models using individual minimum and maximum values for each cell which reduced the mean values to match the 30 km deterministic models; however, to avoid negative values for input parameters such as waterfowl abundance and prevalence, we truncated the triangular distributions to fit within each parameter’s input range (Table 1). Truncating the distributions increased means for each parameter (Table 3, Section B); however, the values were closer to the 30 km deterministic models than the models using a global min/max, and thus used in the final models reported. The most uncertain areas of prediction were located in the western part of the country, and the least uncertain areas were located in the south and east (Figure 4). Models encompassing virus flow from wild birds to poultry (Eq. 4) had CVs that were 40% higher than models describing virus flow from poultry to wild birds (Eq. 3).

**Table 3:** **Comparisons of mean model outputs for 1 km deterministic, 30 km deterministic, and 30 km Monte-Carlo models of H5N1 transmission risk between wild and domestic birds in China**.

(A) Parameter	1 km Deterministic	30 km Deterministic	30 km Monte-Carlo	
**TRUNCATED NORMAL DISTRIBUTIONS**				
*P*_te_ (chickens/km^2^)	379	378	379	
*P*_aq_ (ducks and geese/km^2^)	86	86	86	

**(B) Parameter**	**1 km Deterministic**	**30 km Deterministic**	**30 km Monte-Carlo global min/max**	**30 km Monte-Carlo individual min/max truncated^a^**

**TRIANGULAR DISTRIBUTIONS**				
*W*_EFbr_ (waterfowl/km^2^)	0.01	0.01	0.13	0.03
*W*_EFwi_ (waterfowl/km^2^)	0.006	0.006	0.099	0.037
*P*_te_ × *B*_te_ (chickens/km^2^)	183	184	395	227
*C*_te_ (poultry/km^2^)	1.00	1.00	0.83	0.83

*^a^The 30 km Monte-Carlo individual min/max truncated values were used in the final models, and were closer to the 30 km deterministic means than the models that used a global min/max value for the distributions. (A) Notes values for parameters using truncated normal distributions for the Monte-Carlo simulations. (B) Notes values for parameters using triangular distributions for the Monte-Carlo simulations*.

## Discussion

### Model summary and interpretation

The objective of this study was to lay the foundation for a systematic modeling approach to investigate and predict spatial and temporal patterns of disease transmission risk between poultry and wild waterfowl populations in China. We explicitly took a multi-level approach toward modeling transmission risk between wild and domestic waterfowl in China. The Level 1 deterministic models demonstrated, at a fine resolution, predicted locations of co-occurrence of wild and domestic waterfowl distributions. High risk hotspots during the wintering season were observed in the southern and eastern lowland regions of China (Figure 2). These areas have concentrated poultry populations, particularly free-grazing ducks in association with rice farming, and are important wintering areas for many migratory waterfowl species. Hotspot regions of risk during the breeding season were observed in the northeastern and central-eastern China and had a greater geographic extent but more localized pattern in comparison to the winter risk models. The difference in pattern can be explained in part by waterfowl that tend to breed in the north and high-elevation western regions – areas where wetland habitat is distributed in a patchier, more localized pattern than the extensive lowland wetlands and rice paddies of the southeast (Prosser, in review).

Level 2 and 3 models, although unrealistically simplistic for predicting absolute risk of transmission, takes a first attempt at incorporating H5N1-specific parameters and population numbers for the different bird groups. Virus shedding and uptake rates were included as constants in the model. As our knowledge increases, these constants can be replaced with inputs parameterized to reflect geographic heterogeneity. In particular, the numerator of the uptake term seems unrealistically low (10^−15^). This term was taken directly from a traditional compartmental epidemiological model (37) which is designed to run iterative steps through time. This term has a large affect on the output value of our model, which is represented by a single risk value (in time). Improvements to this term would improve the overall output of our model. In light of these issues, we have used the model to highlight regions of H5N1 transmission risk in relation to other locations.

The effect of the addition of seasonal bird population size was apparent in the model results for QHL and PYL focal areas (Figure 5). Hundreds of thousands of migratory waterfowl return to the PYL region in the winter and reside amongst some of the highest poultry densities in the country. In the QHL region, waterfowl migrate away from the cold and arid plateau for the winter months and risk is lower year-round due to the low poultry densities in the area. Risk for QHL changes during the breeding season (Figure 5C) with the return of tens of thousands of nesting waterfowl to the region. The differences in risk could not be predicted without explicitly incorporating the ecology of the wild bird populations, which is one of the strengths of our approach.

Although waterfowl species were considered as a composite, the contribution of significant species was still evident in certain areas. A concentrated section of risk was observed in northeastern China wintering models (Figures 2 and 3, right panel) which followed the distribution of a single species, the greater white-fronted goose (*Anser albifrons*). The effect of this species was most noticeable in the Level 1 risk models (Figure 2) because input values from the poultry and wild bird populations were given equal weight. The pattern was present in Level 2 models to a lesser degree (Figure 3) since prevalence rates for the greater white-fronted goose were low in comparison to other species (2.2%).

The virus shedding and uptake rates spanned four to ten orders of magnitude. Due to the large range in values, and that we extracted them directly from the literature, we chose to keep these rates fixed in the Monte-Carlo models so we could more clearly assess the effects of our modeled input parameters (wild bird and poultry distributions, and biosecurity and contaminant containment parameters). One term of particular interest was the biosecurity term (*P*_te_ × *B*_te_), which had a substantial effect on the model results. Until data are available to generate a better estimate, we developed two sets of models have been developed for use (28).

Level 3 models showed a similar pattern across China to the deterministic models, confirming that the number of simulations was sufficient for the mean values to converge toward results of the deterministic models (32). This allowed us to identify areas of greatest variability. The lowest predicted errors were in the southeast (Figure 4), which can be explained in part by the high poultry densities in this region (25). The highest predicted errors were in the western regions where waterfowl populations are more localized and sparsely distributed.

### Utility and limits of the models

These models should be considered as a starting point to refine predictions of risk for H5N1 outbreaks. In contrast to existing temporal dynamic models of H5N1 transmission (23, 24, 37), our models focus on transmission risk at the interface of wild and domestic species and illustrate that even simple incorporation of transmission parameters modify the risk map. These are the first spatially based models to incorporate waterfowl distributions from population data. Our approach complements existing dynamic epidemiological models as well as studies which have mapped statistical relationships between outbreak events and environmental or anthropogenic risk factors without having complete information on an entire region or populations (19, 38–42). Our spatial characterization of the susceptible populations and the stepwise examination of the effects of adding model complexity should form a basis for more sophisticated refinement of transmission parameters as these are recognized in other studies.

With growing interest in predicting the risk of future transmission of H5N1 or other emerging strains such as H7N9, all three levels of model have utility. Level 1 models identify areas where transmission can occur due to co-occurrence of wild and domestic species. Level 2 models incorporate population size, prevalence, transmission, and biosecurity parameters. The model structure allows inputs to be changed and new maps to be created. Level 3 models show that the coarser county scale is appropriate for informing surveillance and prevention measures, and is more realistic for assessment. Identification of locations of greater uncertainty in the predictions also helps inform policy decision-making. Applications for the findings of this study may include use by health experts and wildlife officials who are interested in using the poultry to wild risk models (Figure 4A) to identify regions where wild migratory birds are at higher risk of exposure to new and evolving virus strains from poultry. Poultry farmers and health officials may use the wild to poultry risk models to identify areas where farming practices or vaccination programs should be enhanced to protect poultry from exposure to wild birds. As the models take a combined density-dependent and environmental transmission approach, the results may also help target environmental surveillance programs.

**Figure 6:**
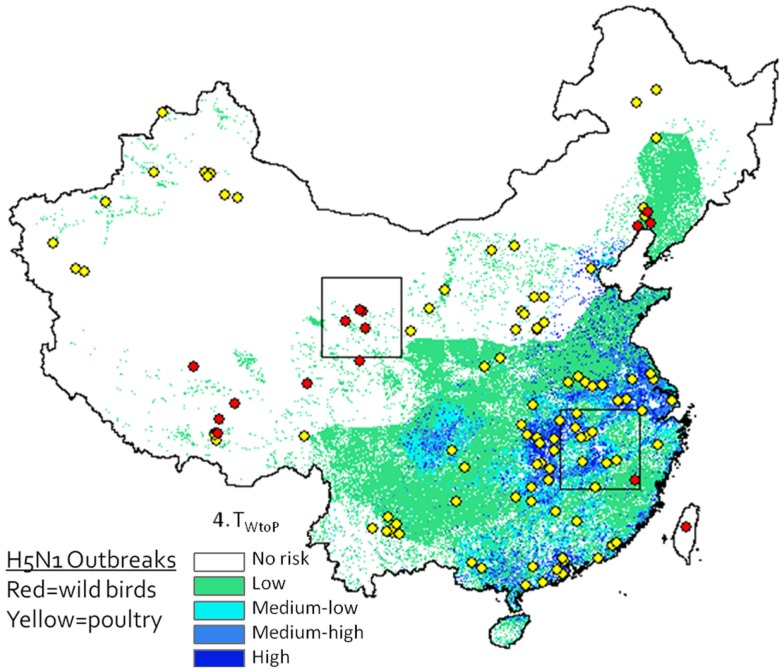
**H5N1 outbreak data (2003–2009) plotted against Level 2 deterministic 1 km resolution H5N1 transmission risk models (Eq. 4)**. Wild bird cases shown by red circles, poultry by yellow circles. Although spatial associations between outbreak data and risk map appear to be high, since the model is intended to predict risk at the interface between poultry and waterfowl, this type of presentation may be misleading as it is not known whether yellow dots represent poultry cases caused by transmission from wild birds (versus poultry) and whether red dots represent wild bird cases caused by poultry (versus wild birds).

A desirable step would be to validate the model predictions using avian surveillance and outbreak data. A strong match exists between outbreak locations and our predicted risk areas (Figure 6), but this may be misleading as our model is intended to predict risk at the interface between poultry and waterfowl, and it is unknown whether these poultry outbreaks were caused by transmission from wild birds (versus poultry) and whether wild bird outbreaks were caused by poultry (versus wild birds). Validation would require geographic and temporal data on infections in wild and domestic birds including information on the type of host that caused the infection. Such detailed surveillance data do not yet exist, and deriving the infecting population from the virus isolates is difficult – even the use of phylogenetic analyses may not definitively answer this question as intermediary transmissions may occur between outbreak events. The spatial and temporal relationships between the wild and domestic waterfowl distributions in our risk models do identify relationships that might guide future targeted, more intensive sampling, and surveillance studies.

Through a structured approach to predicting transmission risk between domestic poultry and wild waterfowl in China, we were able to separate the spatial relationships between poultry and waterfowl from the disease-specific factors to better understand the contributions of each to transmission risk. We explicitly incorporated uncertainty measures with our risk predictions and conducted sensitivity analyses to understand the effects of uncertainty on the model outputs. It is the first analysis of its kind and one of the few that focuses specifically on interactions between the wild and domestic bird populations, providing a unique contribution to our growing knowledge on the topic of wild birds and avian influenza transmission.

## Conflict of Interest Statement

The authors declare that the research was conducted in the absence of any commercial or financial relationships that could be construed as a potential conflict of interest.

## References

[1] Taylor LH Latham SM Woolhouse MEJ Risk factors for human disease emergence. Philos Trans R Soc Lond B Biol Sci (2001) 356(1411):983–9 10.1098/rstb.2001.0888 11516376 PMC1088493

[2] Alexander DJ An overview of the epidemiology of avian influenza. Vaccine (2007) 25(30):5637–44 10.1016/j.vaccine.2006.10.051 17126960

[3] Delgado C Rising consumption of meat and milk in developing countries has created a new food revolution. J Nutr (2003) 133(11):3907S–10 14672289 10.1093/jn/133.11.3907S

[4] Daszak P Cunningham AA Hyatt AD Wildlife ecology – emerging infectious diseases of wildlife – threats to biodiversity and human health. Science (2000) 287(5452):443–9 10.1126/science.287.5452.443 10642539

[5] Brown C Emerging zoonoses and pathogens of public health significance – an overview. Rev Sci Tech (2004) 23(2):435–42 15702711 10.20506/rst.23.2.1495

[6] Morse SS Factors in the emergence of infectious diseases. Emerg Infect Dis (1995) 1(1):7–15 10.3201/eid0101.950102 8903148 PMC2626828

[7] Kovats RS Campbell-Lendrum DH McMichel AJ Woodward A Cox JSH Early effects of climate change: do they include changes in vector-borne disease? Philos Trans R Soc Lond B Biol Sci (2001) 356(1411):1057–68 10.1098/rstb.2001.0894 11516383 PMC1088500

[8] McMichael AJ Global climate change: will it affect vector-borne infectious diseases? Intern Med J (2003) 33(12):552U–4 10.1111/j.1445-5994.2003.00492.x 14656226

[9] McMichael AJ Woodruff RE Hales S Climate change and human health: present and future risks. Lancet (2006) 367(9513):859–69 10.1016/S0140-6736(06)68079-3 16530580

[10] Jones KE Patel NG Levy MA Storeygard A Balk D Gittleman JL Global trends in emerging infectious diseases. Nature (2008) 451(7181):990U–4 10.1038/nature06536 18288193 PMC5960580

[11] World Health Organization Summary and assessment as of 07 June 2013. In: Monthly Risk Assessment Summary: Influenza at the Human-Animal Interface. (2013). Available from: http://www.who.int/influenza/human_animal_interface/Influenza_Summary_IRA_HA_interface_26Apr13.pdf

[12] Xu XY Subbarao K Cox NJ Guo YJ Genetic characterization of the pathogenic influenza A/Goose/Guangdong/1/96 (H5N1) virus: similarity of its hemagglutinin gene to those of H5N1 viruses from the 1997 outbreaks in Hong Kong. Virology (1999) 261(1):15–9 10.1006/viro.1999.9820 10484749

[13] OIE Update on Highly Pathogenic Avian Influenza in Animals: Type H5 and H7. (2013). Available from: http://www.oie.int/animal-health-in-the-world/update-on-avian-influenza/2013/

[14] Imai M Watanabe T Hatta M Das SC Ozawa M Shinya K Experimental adaptation of an influenza H5 HA confers respiratory droplet transmission to a reassortant H5 HA/H1N1 virus in ferrets. Nature (2012) 486(7403):420–8 10.1038/nature10831 22722205 PMC3388103

[15] Russell CA Fonville JM Brown AEX Burke DF Smith DL James SL The potential for respiratory droplet-transmissible A/H5N1 influenza virus to evolve in a mammalian host. Science (2012) 336(6088):1541–7 10.1126/science.1222526 22723414 PMC3426314

[16] Zhu H Wang D Kelvin DJ Li L Zheng Z Yoon SW Infectivity, transmission, and pathology of human H7N9 influenza in ferrets and pigs. Science (2013) 341(6142):183–6 10.1126/science.1239844 23704376

[17] Alexander DJ A review of avian influenza in different bird species. Vet Microbiol (2000) 74(1–2):3–13 10.1016/S0378-1135(00)00160-7 10799774

[18] Webby RJ Webster RG Emergence of influenza A viruses. Philos Trans R Soc Lond B Biol Sci (2001) 356(1416):1817–28 10.1098/rstb.2001.0997 11779380 PMC1088557

[19] Gilbert M Pfeiffer DU Risk factor modelling of the spatio-temporal patterns of highly pathogenic avian influenza (HPAIV) H5N1: a review. Spat Spatiotemporal Epidemiol (2012) 3(3):173–83 10.1016/j.sste 22749203 PMC3389348

[20] Takekawa JY Prosser DJ Newman SH Muzaffar SB Hill NJ Yan B Victims and vectors: highly pathogenic H5N1 and the ecology of water birds. Avian Biol Res (2010) 3:51–73 10.3184/175815510X12737339356701

[21] FAO H5N1 HPAI in different species. Understanding Avian Influenza. (Chap. 3). Animal Production and Health Manual. Rome: FAO (2007). p. 1–15.

[22] Bailey NT Infectious Diseases of Humans: Dynamics and Control. 2nd ed Oxford: Hafner Press, MacMillan Publishing Co. (1975).

[23] Roche B Lebarbenchon C Gauthier-Clerc M Chang CM Thomas F Renaud F Water-borne transmission drives avian influenza dynamics in wild birds: the case of the 2005-2006 epidemics in the Camargue area. Infect Genet Evol (2009) 9(5):800–5 10.1016/j.meegid.2009.04.009 19379841

[24] Rohani P Breban R Stallknecht DE Drake JM Environmental transmission of low pathogenicity avian influenza viruses and its implications for pathogen invasion. Proc Natl Acad Sci U S A (2009) 106(25):10365–9 10.1073/pnas.0809026106 19497868 PMC2690603

[25] Prosser DJ Wu J Ellis EC Gale F Van Boeckel TP Wint W Modelling the distribution of chickens, ducks, and geese in China. Agric Ecosyst Environ (2011) 141:381–9 10.1016/j.agee.2011.04.002 21765567 PMC3134362

[26] Clark L Hall J Avian influenza in wild birds: status as reservoirs, and risks to humans and agriculture. Ornithol Monogr (2006) 2006(60):3–29 10.2307/40166825

[27] FAO Biosecurity for highly pathogenic avian influenza. Animal Production and Health Manual No. 165. Rome: FAO (2008). 80 p

[28] Prosser DJ Wild Birds and Emerging Diseases: Modeling Avian Influenza Transmission Risk Between Domestic and Wild Birds in China [Doctoral Dissertation]. College Park (MD): University of Maryland (2012).

[29] Brewer CA Basic mapping principles for visualizing cancer data using geographic information systems (GIS). Am J Prev Med (2006) 30(2):S25–36 10.1016/j.amepre.2005.09.007 16458787

[30] Brewer CA Pickle L Evaluation of methods for classifying epidemiological data on choropleth maps in series. Ann Assoc Am Geogr (2002) 92(4):662–81 10.1111/1467-8306.00310

[31] Morgan MG Henrion M Uncertainty: A Guide to Dealing with Uncertainty in Quantitative Risk and Policy Analysis. New York, NY: Cambridge University Press (1990). 332 p.

[32] Kroese DP Taimre T Botev ZI Handbook of Monte Carlo Methods. Hoboken, NJ: John Wiley & Sons, Inc (2010).

[33] Delignette-Muller ML Pouillot R Denis JB Dutang C Use of the Package Fitdistrplus to Specify a Distribution from Non-Censored or Censored Data. (2011) [cited 2012 Jun]. Available from: http://cran.r-project.org/web/packages/fitdistrplus/index.html

[34] R Core Development Team R: A Language and Environment for Statistical Computing. Vienna: R Foundation for Statistical Computing (2012). Available from: http://www.R-project.org/

[35] Pouillot R Delingnette-Muller ML Kelly DL Denis JB. The mc2d Package. (2010). Available from: http//riskassessment.r-forge.r-project.org/

[36] Burmaster DE Anderson PD Principles of good practice for the use of Monte Carlo techniques in human health and ecological risk assessments. Risk Anal (1994) 14(4):477–81 10.1111/j.1539-6924.1994.tb00265.x 7972955

[37] Liu R Duvvuri VRSK Wu J Spread pattern formation of H5N1 avian influenza and its implications for control strategies. Math Model Nat Phenom (2008) 3(7):161–79 10.1051/mmnp:2008048

[38] Fang LQ de VlasSJ Liang S Looman CW Gong P Xu B Environmental factors contributing to the spread of H5N1 avian influenza in mainland china. PLoS ONE (2008) 3(5):e2268 10.1371/journal.pone.0002268 18509468 PMC2386237

[39] Gilbert M Xiao X Pfeiffer DU Epprecht M Boles S Czarnecki C Mapping H5N1 highly pathogenic avian influenza risk in Southeast Asia. Proc Natl Acad Sci U S A (2008) 105(12):4769–74 10.1073/pnas.0710581105 18362346 PMC2290786

[40] Martin V Pfeiffer DU Zhou X Xiao X Prosser DJ Guo F Spatial distribution and risk factors of highly pathogenic avian influenza (HPAI) H5N1 in China. PLoS Pathog (2011) 7(3):e1001308 10.1371/journal.ppat.1001308 21408202 PMC3048366

[41] Pfeiffer DU Minh PQ Martin V Epprecht M Otte MJ An analysis of the spatial and temporal patterns of highly pathogenic avian influenza occurrence in Vietnam using national surveillance data. Vet J (2007) 174(2):302–9 10.1016/j.tvjl.2007.05.010 17604193

[42] Tiensin T Ahmed SS Rojanasthien S Songserm T Ratanakorn P Chaichoun K Ecologic risk factor investigation of clusters of avian influenza A (H5N1) virus infection in Thailand. J Infect Dis (2009) 199(12):1735–43 10.1086/599207 19416075

[43] Cao L Barter M Lei G New Anatidae population estimates for eastern China: implications for current flyway estimates. Biol Conserv (2008) 141(9):2301–9 10.1016/j.biocon.2008.06.022

[44] Delany S Scott D Waterbird Population Estimates: Fourth Edition. 4th ed Wageningen: Wetlands International (2006). 239 p

[45] Gaidet N Dodman T Caron A Balança G Desvaux S Goutard F Influenza surveillance in wild birds in Eastern Europe, the Middle East, and Africa: preliminary results from an ongoing FAO-led survey. J Wildl Dis (2007) 43(3):S22–8

[46] Hesterberg U Harris K Stroud D Guberti V Busani L Pittman M Avian influenza surveillance in wild birds in the European Union in 2006. Influenza Other Respi Viruses (2009) 3(1):1–14 10.1111/j.1750-2659.2008.00058.x 19453436 PMC4941908

[47] Kou Z Li Y Yin Z Guo S Wang M Gao X The survey of H5N1 flu virus in wild birds in 14 provinces of China from 2004 to 2007. PLoS ONE (2009) 4(9):e6926 10.1371/journal.pone.0006926 19742325 PMC2735031

[48] Munster VJ Baas C Lexmond P Waldenström J Wallensten A Fransson T Spatial, temporal, and species variation in prevalence of influenza A viruses in wild migratory birds. PLoS Pathog (2007) 3(5):e61 10.1371/journal.ppat.0030061 17500589 PMC1876497

[49] Olsen B Munster VJ Wallensten A Waldenström J Osterhaus AD Fouchier RA Global patterns of influenza a virus in wild birds. Science (2006) 312(5772):384–8 10.1126/science.1122438 16627734

[50] Jeong OM Kim MC Kim MJ Kang HM Kim HR Kim YJ Experimental infection of chickens, ducks and quails with the highly pathogenic H5N1 avian influenza virus. J Vet Sci (2009) 10(1):53–60 10.4142/jvs.2009.10.1.53 19255524 PMC2801098

[51] Shortridge KF Zhou NN Guan Y Gao P Ito T Kawaoka Y Characterization of avian H5N1 influenza viruses from poultry in Hong Kong. Virology (1998) 252(2):331–42 10.1006/viro.1998.9488 9878612

[52] Yu Z Song Y Zhou H Xu X Hu Q Wu H Avian influenza (H5N1) virus in waterfowl and chickens, Central China. Emerg Infect Dis (2007) 13(5):772–5 10.3201/eid1305.061209 17553263 PMC2738434

[53] Chen H Deng G Li Z Tian G Li Y Jiao P The evolution of H5N1 influenza viruses in ducks in southern China. Proc Natl Acad Sci U S A (2004) 101(28):10452–7 10.1073/pnas.0403212101 15235128 PMC478602

[54] Perkins LE Swayne DE Pathogenicity of a Hong Kong-origin H5N1 highly pathogenic avian influenza virus for emus, geese, ducks, and pigeons. Avian Dis (2002) 46(1):53–63 10.1637/0005-2086(2002)046[0053:POAHKO]2.0.CO;2 11924603

[55] Phuong DQ Dung NT Jørgensen PH Handberg KJ Vinh NT Christensen JP Susceptibility of Muscovy (*Cairina moschata*) and mallard ducks (*Anas platyrhynchos*) to experimental infections by different genotypes of H5N1 avian influenza viruses. Vet Microbiol (2011) 148(2–4):168–74 10.1016/j.vetmic.2010.09.007 20943331

[56] Sturm-Ramirez KM Ellis T Bousfield B Bissett L Dyrting K Rehg JE Reemerging H5N1 influenza viruses in Hong Kong in 2002 are highly pathogenic to ducks. J Virol (2004) 78(9):4892–901 10.1128/JVI.78.9.4892-4901.2004 15078970 PMC387679

[57] Sturm-Ramirez KM Hulse-Post DJ Govorkova EA Humberd J Seiler P Puthavathana P Are ducks contributing to the endemicity of highly pathogenic H5N1 influenza virus in Asia? J Virol (2005) 79(17):11269–79 10.1128/JVI.79.17.11269-11279.2005 16103179 PMC1193583

[58] Brown JD Stallknecht DE Swayne DE Experimental infection of swans and geese with highly pathogenic avian influenza virus (H5N1) of Asian lineage. Emerg Infect Dis (2008) 14(1):136–42 10.3201/eid1401.070740 18258093 PMC2600149

[59] Ito T Okazaki K Kawaoka Y Takada A Webster RG Kida H Perpetuation of influenza-A viruses in Alaskan waterfowl reservoirs. Arch Virol (1995) 140(7):1163–72 10.1007/BF01322743 7646350

[60] Lu H Castro AE Evaluation of the infectivity, length of infection, and immune response of a low-pathogenicity H7N2 avian influenza virus in specific-pathogen-free chickens. Avian Dis (2004) 48(2):263–70 10.1637/7064 15283413

